# Diversity of approaches in assessment of executive functions in stroke: Limited evidence?

**DOI:** 10.1016/j.ensci.2015.08.002

**Published:** 2015-08-17

**Authors:** Juliana Conti, Annette Sterr, Sônia Maria Dozzi Brucki, Adriana B. Conforto

**Affiliations:** a Neurostimulation Laboratory, Neurology Clinical Division, Hospital das Clínicas/Sao Paulo University, Brazil; b School of Psychology, University of Surrey, Guildford, UK; c Hospital Israelita Albert Einstein, São Paulo, Brazil

**Keywords:** Stroke, Executive function, Evaluation methodology, Cognition

## Abstract

Stroke is a leading cause of disability worldwide. Cognitive functions and, in particular, executive function, are commonly affected after stroke, leading to impairments in performance of daily activities, decrease in social participation and in quality of life. Appropriate assessment and understanding of executive dysfunction are important, firstly to develop better rehabilitation strategies for executive functions per se and secondly to consider executive function abilities on rehabilitation strategies in general. The purpose of this review was to identify the most widely used assessment tools of executive dysfunction for patients with stroke, and their psychometric properties.

We systematically reviewed manuscripts published in English in databases from 1999 to 2015. We identified 35 publications. The most frequently used instruments were the Stroop, Digit Span and Trail making tests. Psychometric properties were described for the Executive Function Performance Test, Executive Clock Drawing Task, Chinese Frontal Assessment Battery and Virtual Action Planning — Supermarket, and two subtests of the Cambridge Cognitive Examination — Revised.

There is a paucity of tools to reliably measure executive dysfunction after stroke, despite the fact that executive dysfunction is frequent. Identification of the best tools for executive dysfunction assessment is necessary to address important gaps in research and in clinical practice.

## 1 Introduction

Stroke is characterized by fast and focal development of neurologic symptoms that reflect loss of brain function due to ischemia or hemorrhage [1], [2]. Strokes were responsible for 10% of all world deaths and 4% of loss of disability-adjusted life years in 2010 [3]. While age-standardized incidence of stroke significantly decreased by 12% in high-income countries, it increased by 12% in low-middle income countries between 1990 and 2010 [3]. Also, the number of stroke survivors increased by 84% in low- and middle-income countries between 1990 and 2010 [3].

More than two-thirds of patients with stroke have limitations to live independently. Stroke can cause a catastrophic impact in patients' lives, due to impairments in physical and psychological functions, as well as in cognitive, perceptual and communication skills [4]. Burden is not only caused by the direct deficits caused by acute stroke, but also by dementia. In a recent meta-analysis, rates of dementia after stroke varied from 9.1% (in population-based studies) to 14.4%, (in hospital-based studies) [5].

Cognitive dysfunction strongly contributes to disability and loss in quality of life. It can also often be a barrier for returning to work. Because cognitive impairments are ‘invisible’, patients have less awareness of them and it is more difficult to recognize the deficits in the workplace so that the necessary adjustments can be made. Studies in post-stroke patients often report scores in the Mini-Mental State Examination (MMSE) for cognitive evaluation, but this test is insensitive to detect executive dysfunction and does not capture more subtle deficits in cognition [5].

When a comprehensive assessment of cognition was performed in a multicenter study in Belgium and the Netherlands, dysfunction was present in 55% (89/190) of individuals after stroke. The following functions were compromised: executive function (39.1%), visual perception and construction (38.1%), neglect (31.3%), abstract reasoning (25.6%), verbal memory (25.6%), language (25.6%) and visual memory (22.0%) [6]. Another study from New Zealand showed that 30–50% of 307 patients had impaired cognitive performance [7]. The most common deficit was executive dysfunction (30.4%). The prevalence of executive dysfunction after stroke ranges from 18.5% to 39%, depending on definitions and instruments used for its evaluation [6], [8], [9].

Executive function involves planning, problem solving, dealing with new situations, decision-making and performing complex tasks [10]. These functions are part of the cognitive process of acquiring, keeping and applying knowledge to behavior [11]. Theories about executive function have been proposed: (1) Single system: believes that injury of a single executive function process leads to impairment; (2) Construct-led: working-memory and fluid-intelligence are the most important functions; (3) Multiple process: Executive functions are composed of different functions and processes working together during daily activities, but it is possible to evaluate each function separately; and (4) Single-symptoms: there are two symptoms that are common in patients with EF deficits: confabulation (characterized by impairment in memory control) and multitasking (patients with impairments in higher-level functions, leading to a problems in organizing/planning daily routines) [11]. Executive function roles can also be divided into: (1) shifting: the capacity to initiate different tasks at the same time and return to each one; (2) updating: to monitor information and organize it according to a different objective, and retrieve it when necessary; and (3) inhibition: to inhibit one stimulus and focus on a task or problem [12]. These three functions are connected and can interfere with one another. Moreover, they contribute to performance of more complex executive functions and influence rehabilitation outcomes [12], [13].

We conducted a review of the literature about tools for assessment of executive functions in stroke to identify tests performed in common practice worldwide and to determine the most appropriate evaluation instruments according to their psychometric properties, using an evidence-based approach.

## 2 Methods

This study was conducted according to the Preferred Reporting Items for Systematic Reviews and Meta-Analyses — PRISMA [14]. One investigator reviewed the manuscripts. Manuscripts published in English in Medline, Pubmed and Google Scholar from 1999 to 2015 were searched using the terms “stroke” and each of the following: executive function, executive dysfunction, working memory, rehabilitation or cognition. During the research we also considered the title, key word and abstract of the papers before including them in this review. Only original papers were considered.

We only included studies in which the primary goal was assessment of executive dysfunction in adults (18-90 years). Abstracts that did not specifically report the assessment of executive function or executive dysfunction, or in which executive function or dysfunction were not the primary objective, were excluded. Abstracts reporting on a mix of neurological disorders rather than stroke specifically were also not included. For those abstracts fulfilling the inclusion criteria, the full manuscripts were obtained to extract the methodological details listed below. In addition to the manuscripts identified by the key word search, we also included papers cited in other systematic reviews or meta-analysis.

We checked frequencies of use of the different tools of evaluation across studies. In addition, tools for evaluation of executive dysfunction were grouped in tables according to time from stroke described in the studies: less than one month, one-six months and six months or more. Table 1, Table 2, Table 3, Table 4 describe: (1) references, including authors and country of research; (2) instruments used for executive function assessment; (3) objective; (4) sample characteristics, taken from the methods sections of the original papers; including whether or not performance of patients was compared to performance of controls and if the lesion site was described; (5) psychometric data of the instruments: internal consistency (Cronbach's α), reliability (test–retest and inter-rater; intraclass correlation coefficient, ICC, or Pearson's r), concurrent validity (evaluated with Spearman's rho or Pearson's r) [15], and (6) main results: the relation between executive dysfunction and other conditions (such as depression or motor function). Table 5 describes the abbreviations of the tools listed in Table 1, Table 2, Table 3, Table 4.

**Table 1:** Assessment tools for executive dysfunction, evaluated in patients < 1 month after stroke.

Article, country	Tools	Objective	Sample characteristics	Psychometric Data	Main Results
[18] Brazil	DS, VFT and TMT	Relation between depression-executive dysfunction syndrome and patients with stroke	First-ever ischemic stroke (n = 87), with subgroups: older (> 60 y; n = 62) and younger (≤ 60 y; n = 25) Stroke affecting the LCSPT (n = 27) or not (n = 48)	Not reported	Depression-executive dysfunction syndrome significantly more frequent in younger than in older subgroup
[16] France	DS, VFT, TMT, M-WCST, Tower of London Test and Stroop Test	Relation between working memory and EF	Frontal strokes (n = 17, mean age, 47 y); “posterior” strokes (n = 12, mean age, 43.3 y) CG (n = 29, mean age, 46.3 y)	Not reported	Both groups had impaired working memory
[23] United States	TMT and DS	Effects of stroke on TMT and DS performance. Determine whether patients with frontal lesions have poorer performance on TMT B and DS than patients with non-frontal brain lesions	TMT: non-frontal lesions (n = 122) and frontal lesions (n = 4). DS: Non-frontal (n = 175) and frontal lesions (n = 52).	Not reported	TMT A and B as well as DS forward and backwards scores similar for patients with frontal and non-frontal lesions; no relations between test performance and stroke severity
[26] Sweden	EFPT and AMPS	Concurrent validity	Stroke (n = 23)	Concurrent validity: rho = 0.61	Subtests: Cooking Task: ICC = 0.54, Paying bills task: ICC = 0.57, Medication task: ICC = 0.56 and Telephone task: ICC = 0. All tasks of EFPT: rho = 0.60
[31] United States	CLOX	CLOX divided in two parts: CLOX 1 (free draw of a clock) and CLOX 2 (copy of a clock)	Stroke (n = 66, mean age, 58.8 y) Right hemisphere (52%), left hemisphere (30%), bilateral (8%), not defined (11%)	Test–retest reliability: CLOX 1, r = 0.62.CLOX 2, r = 0.68.	–
[82] USA	Sorting Test, Color–Word Interference, TMT and EFPT	Determinate the presence of ED immediately after mild stroke	Mild stroke (n = 53, mean age, 56.2 y) 1 week after stroke	Not reported	66% of the subjects had poor performance in 1 out of 4, and 27% in 2 or more out of 4 measures of executive function
[46] Poland	TMT, VFT, Go–No Go Task WCST	Investigate the effect of lesion side (left/right) and location (anterior/posterior) on WCST scores	Unilateral ischemic stroke (n = 44, mean age, 56 y)	Not reported	Worse performance in patients with frontal lesions
[35] The Netherlands	ST and Concept Shifting Test (CST)	Temporal relation between depressive symptoms and executive dysfunctions	First-ever unilateral stroke (n = 116, mean age, 65.8 y)	Not reported	Depression and ED occurred in 22% of patients after 1 month. 33% of the patients with depression and ED still had symptoms after 2 years
[28] United States	EFPT	If the components of the EFPT are sensitive to impairments in executive abilities	First-ever strokes (n = 20, mean age, 58.8 y)	Inter-rater reliability: ICC = 0.91 Internal Consistency: α = 0.94	–
[25] China	CFAB, ST, M-WCST, VFT and Go–No Go Task	Correlation between executive function and emotional incontinence	Stroke (n = 39, mean age, 63.8 y) CG (n = 39, mean age, 64.4 y)	Not reported	Emotional incontinence was associated with frontal or basal ganglia lesions.
[40] United States	WCST, DS andDKEFS	Frequency of ED.Relation between ED, stroke severity and premorbid risk factors	Stroke (n = 47, mean age, 65.8 y) TIA (n = 9, mean age, 64.1 y) CG (n = 10, mean age, 58.5 y)	Not reported	Impaired EF in 50% of subjects with stroke or TIA. Cognitive impairment was not related to stroke severity

EF = Executive Functions. ED = Executive Dysfunctions. LCSPT = inside the limbic–cortical–striatal–pallidal–thalamic circuit. n = number of subjects. CG = Control Group. TIA = Transient Ischemic Attack. y = years.

**Table 2:** Executive Function assessment tools for executive dysfunction, evaluated in patients, 1–6 months after stroke.

Ref.	Tools	Objective	Sample characteristics	Psychometric data	Main results
[17] Finland	WCST,DS,TMT, VFT, and ST	If the association of depression and ED increases the chances of a recurrent ischemic stroke	First-ever ischemic stroke (n = 223, mean age, 71 y) Only 205 performed EF evaluation	Not reported	83/205 (40%) presented ED. The mean time until the first recurrent stroke was shorter for patients with depression and patients with depression-executive dysfunction syndrome
[42] South Korea	VFT, DS and ST	Investigate the patterns of the neuropsychological deficits, including EF	Posterior cerebral artery (PCA) strokes (n = 12, mean age, 68.5 y)	Not reported	Stroke in the PCA territory was frequently associated with ED. Extended lesions into the splenium of the corpus callosum and posterior ventral temporal lobe were associated with greater cognitive impairment
[19] Finland	WCST,DS,TMT and ST	Investigated the influence of post stroke depression and related factors on survival 3 months post stroke	Stroke (n = 257, mean age, 71.9 y)	Not reported	ED was present in 114/257 (44.4%) patients and was associated with shorter survival. ED + depression were also associated with shorter survival
[48] Finland	WCST,DS, VFTTMT and ST	Patients with ED would have more often brain infarcts affecting the frontal–subcortical–circuit and more extensive white matter changes	Stroke (n = 214, age range, 55–85 y)	Not reported	Number of infarcts in left hemisphere was higher in patients with ED. ED was presented in 73 (34.1%). 21/73 had infarct in the pons. Moderate to severe white matter changes were often seen in patients with ED ED was associated with lesions of the frontal–subcortical circuit
[33] Finland	WCST,DS, VFTTMT and ST	Depression-dysexecutive syndrome (DES) might be related to frontal–subcortical circuit dysfunction	Ischemic stroke (n = 158, age range, 55–85 y) CG (n = 28, mean age, 67 y)	Not reported	53/158: presented ED 21/158 had DES; they showed significantly more brain infarcts affecting frontal–subcortical circuits, and also coped less well with complex activities of daily living
[47]. Finland	WCST,and ST	Examine EF	Ischemic stroke (n = 256, mean age, 71.1 y)	Not reported	Frequency of EF was 40.6% (n = 104). Patients with ED more often presented the following symptoms: low levels of education, poor performance in ADLs, cognitive impairment and dementia
[30] United Kingdom	CAMCOG-R, Weighl and Raven tests	Evaluate the concurrent validity: EF tests of the CAMCOG-R compared with The Weighl and Raven tests	Stroke (n = 83, mean age, 75 y)	Weighl: r = 0.46; Raven: r = 0.59	–
[41] Finland	TMT, ST, WCST and VFT	If frontal stroke causes ED or slowing of mental processing	Ischemic Stroke: frontal (n = 62, mean age, 70.9 y); Non-frontal (n = 188, mean age, 70.3 y);CG (n = 39, mean age, 66.5 y)	Not reported	EF was impaired in both frontal and non-frontal groups. Mental processing was more impaired in the frontal group.

CG = Control Group. I = Ischemic. F = frontal; NF = Non-frontal. EF = Executive Functions. ED = Executive Dysfunctions. y = years. n = number of subjects.

**Table 3:** Function assessment tools for executive dysfunction, evaluated in patients ≥ 6 months after stroke.

Ref.	Tools	Objective	Sample characteristics	Psychometric data	Main results
[84] Canada	TMT, DS	Exercises and recreation could improve EF in adults with chronic stroke compared with a delayed intervention	Stroke intervention (INT, n = 12) or delayed intervention (D-INT, n = 16)	Not reported	Compared with the D-INT group, the INT group significantly improved selective attention and conflict resolution (P = .02), working memory (P = .04) at the end of the 6-month intervention period.
[29] USA	DKEFS, Color–Word interference subtest, TMT and Letter–Number Sequencing	Evaluate the association between executive function and coping strategies	Stroke (n = 15, mean age, 60 y) 27% right stroke 73% left stroke	Not reported	ED was not related to active coping
[32]. Israel	VAP-S, BADS, Observed Tasks of Daily Living-Revised (OTDL-R)	Construct and Concurrent validity	Right hemispheric (n = 12)Left hemispheric (n = 11) Bilateral stroke (n = 1) (Mean age, 58.9 y)CG (n = 24, mean age, 60.1 y)	VAP-S (number of purchases) and: BADS, r = 0.42; OTDL-R, r = 0.64 VAP-S (correct actions) and: BADS, r = 0.61; OTDL-R, 0.68 OTDL-R and BADS: r = 0.72	Worse performance in patients than in controls in: number of purchases and correct actions of the VAP-S; rule shift card, key search and modified 6-element tests of the BADS
[83] The Netherlands	DS	Associative working memory task is sensitive in stroke patients. Also, investigate the role of long-term encoding in relation to working memory.	Stroke (n = 24, mean age:52.1 y) and CG (n = 31, mean age: 50.6 y)	Not reported	The binding condition was more difficult than both single-feature conditions, but patients performed equally well as compared to matched healthy controls. No deficits were found on the subsequent long-term memory task. Associative working memory may be mediated by structures of the medial temporal lobe.
[21] Ireland	TMT, ST, Zoo Map (subtest of the BADS), Frontal Assessment Battery and DS Backwards	How EF may affect performance on basic and complex 10 − meter gait tests	Stroke (n = 20, mean age, 69.1 y) Able to walk with or without an assistive device	Not reported	Correlation between dysexecutive function and poor performance in the complex 10-meter gait test
[27] United States	DS Backwards and Flanker Test	Relation between improvement in aerobic fitness and changes in cognition and EF	Stroke (n = 9, mean age, 63.7 y)	Not reported	Significant improvement in DS-B and FT performance after 12 weeks of the exercise program
[20]. Canada	TMT, ST and DS Backwards	Exercises or practicing motor (included stretching, balance, and task-specific exercises) and recreation to improve EF and memory	Stroke (n = 11, mean age, 67 y) Lower Limb. Able to walk 3 m with or without an assistive device	Not reported	Significant improvement in the DS–B and ST after 6 months of the program of exercises and recreation
[22] United States	EFPT, VTF, TMT, DS and Functional Independence Measure (FIM), among others	Examine the reliability and validity of the EFPT	Stroke (n = 73); Mild (NIHSS = 2, n = 59, mean age, 64.5 y); Moderate (NIHSS = 10.6, n = 14, mean age, 64.1 y); CG (n = 22, mean age, 59.4 y)	Inter-rater reliability: ICC = 0.91; Internal Consistency: α = 0.94; Concurrent validity: ATF (r = –0.47), TMT B (r = 0.39), DS Backward (r = – 0.49); FIM (r = − 0.40)	Subtests: Cooking Task: ICC = 0.94; Paying bills task: ICC = 0.89; Medication task: ICC = 0.87; Telephone task: ICC = 0.79. Construct validity: Performance of patients with moderate stroke was worse than performance of patients with milder stroke and even worse than of controls
[38] Australia	BADS and Tinkertoy Test	Performance in measures of executive functions employment and productivity outcomes	Stroke (n = 27, mean age, 47.3 y)	Not reported	10 patients returned to work in the 12-months follow-up and 17 did not. The employed group performed better on the EF tests than the unemployed group
[37] Canada	Stroop Test (ST) and Digit Span test (DS)	Executive-controlled processes, performance in balance and mobility	Stroke (n = 63, mean age, 65 y) Able to walk for 10 m independently	Not reported	Significant association between EF and balance
[34] United Kingdom	Wisconsin Card Sorting Test (WCST), andControlled Oral Word Association (COWA)	Examine the effect of antidepressants (nortriptyline and fluoxetine) on post-stroke EF	Stroke (n = 30, mean age, 65.5 y) (nortriptyline or fluoxetine groups) Placebo (n = 17, mean age, 71.7 y)	Not reported	Both groups showed an improvement in EF after 21 weeks of treatment. Placebo group showed a decline in EF after 21 weeks

CG = Control Group. EF = Executive Functions. ED = Executive Dysfunctions. IADL = Instrumental Activities of Daily Living. NIHSS = National Institute of Health Stroke Scale. y = years. n = number of subjects.

**Table 4:** Function assessment tools for executive dysfunction, evaluated in patients — time from stroke not specified.

Ref.	Tools	Objective	Sample characteristics	Psychometric data	Main results
[39] Australia	BADS and TMT	Investigate the relation between executive functions and driving performance	Stroke (n = 19, mean age, 70.1 y) CG (n = 22, mean age, 64 y)	Not reported	Moderate correlation between the TMT B and the driving score test (rho = 0.34) Control group performed better than stroke group in driving assessment
[45] United States	Complex Task Performance Assessment (CPTA), DKEFS, M-WCST, VFT and TMT	Evaluation of dysexecutive syndrome with the CPTA	Stroke (n = 6, mean age, 55.7 y) CG (n = 4, mean age, 55.7 y)	Not reported	Stroke group performed worse in the CTPA then the CG
[24] China	WCST, CFAB and Initiation–perseveration subtest of Mattis	Evaluate validity and reliability of the CFAB	Small subcortical infarct(n = 31, mean age, 73.5 y) CG (n = 41, mean age, 69.6 y)	CFAB: Internal consistency: α = 0.77 Inter-rater reliability: rho = 0.85 Test–retest reliability: rho = 0.89 Concurrent Validity: r = 0.63	Patients performed worse in the Mattis and the WCST, as well as in fluency, motor series and go–no-go items of the CFAB
[43] The Netherlands	WCST, VFT, ST, DS, TLT	Thalamic structures have specific roles in each of these functions: memory, executive functioning and attention	Stroke (n = 22, age between: 22 to 83 y) Thalamic infarction (3 months to 24 years of lesion)	Not reported	Thalamic structures are involved in memory, executive functioning and attention
[44] United States	ST and CCST	Evaluated ED could be found in non-demented patients with subcortical lacunar lesions	Subcortical lacunar lesions. (n = 39, mean age, 73.7 y) CG (n = 27, mean age, 72.8 y)	Not reported	Stroke patients performed worse in ST and CCST. Worse performance associated with more extensive lacunar lesions

CG = control group. EF = Executive Functions. ED = Executive Dysfunctions. y = years. n = number of subjects.

**Table 5:** Tests and abbreviations.

Most cited test's name	Abbreviations
Assessment of Motor and Process Skills	AMPS
Behavioral Assessment of Dysexecutive Syndrome	BADS
Cambridge Cognitive Examination — Revised	CAMCOG-R
Chinese Frontal Assessment Battery	C-FAB
Color–Word interference	CWI
Controlled Oral Word Association	COWA
Complex Task Performance Assessment	CTPA
Concept Shifting Test	CST
Delis–Kaplan Executive Function System	DKEFS
Digit Span	DS
Executive Clock Drawing Task	CLOX
Executive Function Performance Test	EFPT
Frontal Assessment Battery	FAB
Initiation–perseveration subtest of Mattis	IPS-Mattis
Letter–Number Sequencing	LNS
Modified Wisconsin Card Sorting Test	M-WCST
Observed Tasks of Daily Living-Revised	OTDLR
Reven test	RT
Stroop Test	ST
Tinkertoy Test	TT
Trail making	TMT
Tower of London Test	TLT
Verbal Fluency Test	VFT
Virtual Action Planning — Supermarket	VAP-S
Weigl test	WT
Wisconsin Card Sorting Test	WCST

## 3 Results

Initially, 210/997 abstracts were identified and 35/210 manuscripts were selected for this review (Fig. 1).

**Fig. 1:**
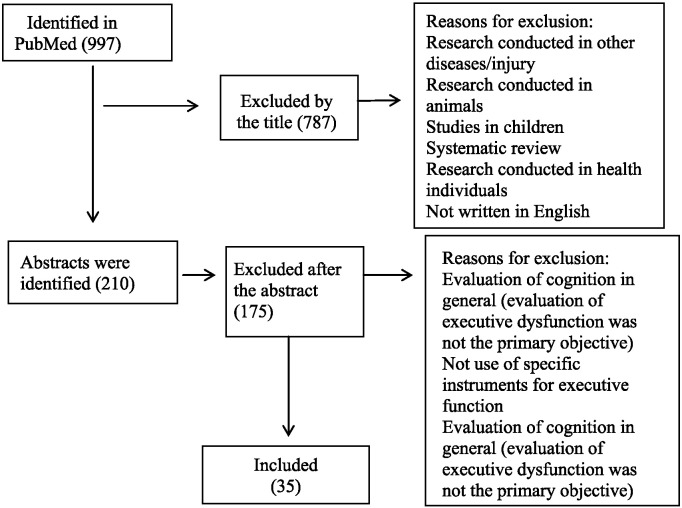
Flow diagram: identified manuscripts and reasons for exclusion.

The results of 35 papers were organized according to time from stroke: Table 1 (up to one month, 11 studies), Table 2 (one-six months; eight studies) and Table 3 (6 months and more, eleven studies). Five manuscripts did not specify the period of evaluation after stroke onset (Table 4).

The tools most frequently used in the 35 studies identified were: Trail making Test (17) Stroop Test (15), Digit Span (15), Wisconsin Card Sorting Test (11), Verbal Fluency Test (11), and Behavioral Assessment of Dysexecutive Syndrome (4). In addition, most of the papers used more than one tool to evaluate executive functions. The most combined used instruments were: Trail Making Test, Digit Span and Verbal Fluency [16], [17], [18], Digit Span, Trail making and Stroop tests [19], [20], [21]; Digit Span tests [22], [23].

Modified versions of tests were reported in 6/35 manuscripts: Chinese Frontal Assessment Battery [24], [25] Modified Wisconsin Card Sorting Test [16], [17], [25] and Swedish version of the Executive Function Performance Test [26].

One or two subtests of the following instruments of evaluation were applied in 7/35 studies: Initiation-perseveration subtest of the Mattis Dementia Rating Scale [24]; Zoo map, from the Behavioral Assessment of Dysexecutive Syndrome [21]; Digit Span Backwards [21], [27]; Color–word Interference subtest [28], [29] and Executive Functions subtests of the Cambridge Cognitive Examination — Revised [30]. These subtests were used in addition to other tests to evaluate executive dysfunction.

Detailed psychometric properties of the Executive Function Performance Test were described for the English (six months or more after stroke and up to one month after stroke) and Swedish (up to one month after stroke) versions [22], [26], [28]. This test includes the following tasks: simple meal preparation, making a telephone call, paying the bills and taking medication. Therefore, it evaluates initiation, organization, sequence, safety and judgment, and conclusion of the task. The test had excellent inter-rater reliability (ICC = 0.91) [22], [28]. Internal consistency was also excellent (α = 0.94) [15].

Concurrent validity was poor when Executive Function Performance Test scores were correlated with scores in the Animal Fluency, Trail Making Test-B, Digit Span tests and the Functional Independence Measure [22], and moderate when correlated with the Assessment of Motor and Process Skills [26].

Psychometric properties of other executive dysfunction tests, listed below, were reported in four more studies (Table 1, Table 2, Table 3, Table 4):

• Cambridge Cognitive Examination — Revised [30] (Table 2): This is a brief neuropsychology battery subdivided into eight subscales. It evaluates the following cognitive domains: orientation (time and place), language (comprehension and expression), memory (incidental, remote, recent and new learning), attention, calculation, praxis, perception and executive functions (abstract thinking, ideational fluency and visual reasoning). The instrument comprises a questionnaire as well as tasks. Leeds and colleagues described properties of two executive functions subtests (ideational fluency and visual reasoning). Concurrent validity was reported to be poor to moderate with the Weigl (r = 0.46) and Raven (r = 0.59) tests.
• Executive Clock Drawing Task [31] (Table 1). The Executive Clock Drawing Task is divided into two parts: In Executive Clock Drawing Task 1, the patient is instructed to draw a clock on the back of the Executive Clock Drawing Task form. Executive Clock Drawing Task 2 consists of a simple copying task. The test–retest reliability was reported to be moderate (r = 0.62).
• Chinese Frontal Assessment Battery [24] (Table 4), a bedside cognitive screening divided in six items that evaluate six executive domains conceptualization, mental flexibility, programming, sensitivity to interference, inhibitory control, and environmental autonomy. Moderate to very good psychometric properties were reported for the version in Chinese: for internal consistency, α = 0.77 and for inter-rater reliability, r = 0.89. Concurrent validity was evaluated by correlations between Chinese Frontal Assessment Battery scores and the number of categories completed (r = 0.45), and the number of perseverative errors (r = − 0.37) of the Wisconsin Card Sorting Test.
• Virtual Action Planning — Supermarket [32] (Table 3), a virtual supermarket that evaluates the ability to buy seven items. The score is based on eight variables (total distance, total time, number of total items purchased, number of correct actions, number of incorrect actions, number of pauses, the combined duration of pauses and time to pay). Poor to moderate correlations were reported in the Hebrew version of the Virtual Action Planning — Supermarket (number of purchases) and the Behavioral Assessment of Dysexecutive Syndrome (r = 0.42) or the Observed Tasks of Daily Living-Revised (r = 0.64). Moderate correlations were reported between the Virtual Action Planning — Supermarket (correct actions) and the Behavioral Assessment of Dysexecutive Syndrome (r = 0.61) or the Observed Tasks of Daily Living-Revised (r = 0.68).

Relations between executive dysfunction and other conditions were discussed in 15/35 manuscripts: depression [17], [18], [19], [33], [34], [35], emotional incontinence [25], motor impairment and rehabilitation [20], [21], [27], [36], [37], coping [29], employment/productivity outcomes [38] and driving performance [39]. Altogether, patients with executive dysfunction presented poor performance on physical tasks often had depression and difficulties to return to a productive life. Long-term antidepressant treatment improved executive function in patients with stroke [34]. Only a few studies mentioned effects of antidepressants in their studies [18], [35]. On the other hand, executive dysfunction was not significantly correlated with emotional incontinence or coping after stroke.

Executive dysfunction was compared in patients with stroke and in controls in 12/35 manuscripts: three, less than 1 month after stroke [16], [25], [40]; two, one-six months [33], [41]; four, six months or more [22, 32,34, 51]; four, time from stroke not specified [24], [39], [44], [45]. Overall, as expected, executive function was worse in patients than in controls [16], [22], [24], [25], [32], [39], [40], [41], [44], [46].

Lesion sites were described in only 11/35 manuscripts: frontal or non-frontal [23], [41]; frontal–subcortical circuit [48]; cortical or subcortical territory of the posterior cerebral artery [42]; “posterior region” [16]; right, left or bilateral stroke [31], [32]; right and left side [29]; stroke inside the limbic–cortical–striatal–pallidal–thalamic circuit (LCSPT) [18], subcortical lacunes [44] and thalamic infarcts [43]. The latter study revealed an association between lesions in the LCSPT circuit and depression, but not executive dysfunction, in patients over 60 years [18]. Three manuscripts investigated executive dysfunction in patients with frontal or non-frontal lesions [23], [41]. Differences between these two groups of patients were found in two studies [16], [41]. Lesions in the territory of the posterior cerebral artery were frequently associated with executive dysfunction [42]. In addition, executive dysfunction was associated with lesions in the frontal–subcortical circuit [48].

## 4 Discussion

The present study demonstrates that, in contrast to the extensive literature on motor function after stroke, the number of studies specifically evaluating executive function/dysfunction in stroke is relatively small (N = 35). These studies used a diverse range of assessment tools, and only a few provided adequate information on the psychometric properties of the tests employed. Literally all studies were performed in high-income, mostly western countries, despite the fact that most strokes occur in low- and middle-income countries. The study further shows that various assessment tools were used for different stages of recovery. And while most studies reported time since stroke at the point of assessment, the evidence-base available does not specifically address how stroke chronicity might have affected test performance. Several papers further highlighted the shortcomings of the evidence base concerning the association between executive dysfunction and other types of impairment, as well as rehabilitation interventions [18], [19], [20], [21], [25], [29], [34], [35], [37]. Moreover, more studies with larger sample sizes are necessary to improve the evidence base, and indeed, clinical practice in the area of executive function in stroke.

The most frequently reported tools for assessment of executive function were the Trail Making Test, Stroop test, and Digit Span tests. They evaluate different aspects of executive functions. Digit Forward is a measure of attention and immediate memory, while Digit Backwards is related to more complex attention and working memory. Trail Making Test A is related to processing speed and flexibility, while Trail making B is a measure of mental flexibility, sustaining, shifting and dividing attention. The Stroop test is used to verify inhibitory control. Together these tests therefore provide an easy and quick to administer, yet relatively good estimate of executive function. However, despite the practical advantageous of those tests in clinical settings, their psychometric properties were not yet characterized in patients with stroke.

Psychometric properties of the Stroop test and the Trail Making Test were described for healthy subjects in different languages, including English [49], [50], Swiss-German [51], Portuguese [52], [53], and Italian [54]. Psychometric properties of the Trail making test were further described for patients with brain damage in various languages [55], [56]. Future research is required to define internal consistency, inter-rater and test–retest reliabilities, as well as discriminating, construct and concurrent validities of these instruments in patients with stroke, at different stages of recovery and with particular lesion locations. Moreover, it is important to consider the influence of motor or language impairment on test performance as well as the influence of educational level. The latter is particularly important in low-income countries where reading and writing skills are often quite poor.

Psychometric properties in patients with stroke were comprehensively described for the Executive Function Performance Test. This test had excellent inter-rater reliability and internal consistency. It was compared with measures of executive functions (Animal Fluency, Trail making Test-B, Digit Span tests and the Functional Independence Measure) [22], as well as performance-based tools for Activities of Daily Living and executive functions (Assessment of Motor and Process Skills) [26]. Concurrent validity was found to be moderate.

Besides the Executive Function Performance Test, psychometric properties were described for only four other instruments in patients with stroke. Test–retest reliability was reported to be moderate for the Executive Clock Drawing Task and was not described for other tests. Inter-rater reliability was excellent for the Chinese Frontal Assessment Battery, but not reported in other tests. Internal consistency was very good for the Chinese Frontal Assessment Battery. Moderate concurrent validities were described for executive functions subtests of the Cambridge Cognitive Examination — Revised [20], Chinese Frontal Assessment Battery [24] or Virtual Action Planning — Supermarket [32] and other tests.

As the discussion above highlights, psychometric data for executive function tests in stroke are insufficient, and this is a recognized demand. The NIH EXAMINER represents a new test battery for executive functions for neurological disease which has recently been validated in a multicentre study validated with 1248 participants (included 485 participants below the age of 18 years and 763 participants 18 years and older) [57], [58], [59]. However, patients with stroke were not included. Considering the impact of executive dysfunction in patients with stroke, we suggest that this instrument should be translated, validated and adapted to different cultures worldwide.

Executive function is a complex cognitive domain that influences and is influenced by other human functions such as behavior and emotional, motor and other cognitive domains. Executive dysfunction can compromise functional status, due to the interaction with other conditions such as depression [18], [19], [34], [35], emotional incontinence [25], motor impairments [20], [21], [27], [36] and driving performance [39]. In addition, impairment in these conditions can lead to poorer quality of life. Moreover, executive function can interfere in daily routine, including the capacity to deal with unfamiliar situations in new environments.

In particular, executive function is frequently impaired in elderly patients with depression [60], and is a predictor of depression [61], [62]. Even after treatment of depression, impairments in executive functions can persist and are associated with worse outcomes [60]. Depression is common after stroke, with prevalence ranging from 25% to 70% in all survivors [63]. Therefore, patients may simultaneously present executive dysfunction and depression (co-morbid prevalence of 18.3%) [17], [18], [19], [33], [61]. Considering that depression and executive dysfunction interact with each other and are associated with poor outcomes after stroke, studies that report performance in executive function in patients with stroke should also investigate symptoms of depression.

Not only depression interferes on executive dysfunction after stroke, but also medication used for treatment may interfere with performance of the patient [64]. For this reason, medication should be considered during evaluations in clinical practice and in research studies in general; however not all studies described medications used.

In addition, not only the stroke lesion itself, but also vascular cognitive impairment can contribute to executive dysfunction [65]. Vascular cognitive impairment in non-demented patients is characterized by cognitive decline secondary to cerebrovascular disease and can present with executive dysfunction [66], [67], [68]. The rate of vascular cognitive impairment ranges from 10.5 to 37% of patients without stroke or dementia [69], [70], [71]. In Brazil, for instance, vascular cognitive impairment in patients with ischemic stroke was diagnosed in 16.8% of 172 subjects [72]. It is important that studies about executive function in patients with stroke define whether vascular cognitive impairment was an exclusion criterion.

Finally, details about lesion sites were scarce or absent in most studies that reported evaluation of executive dysfunction in patients with stroke. It is desirable that more information about lesion location is provided in future studies. Two studies used magnetic resonance imaging (MRI) for lesion characterization and compared the result with the measures for executive dysfunction [33], [48]. Patients with executive dysfunction presented infarcts in the frontal–sub cortical circuit [48]. In addition, the aging brain suffers changes such as white matter lesions and microbleeds, which are associated with cognitive and functional decline [67].

Stroke is not the only cerebrovascular diseases that can cause cognitive impairment. White matter lesions [73] and cerebral microbleeds [74] may play an important role. White matter lesions are prevalent in people over 60 years, can be detected in up to 90% of neurologically symptom-free elderly and are associated with cognitive impairment [75], [76]. Cerebral microbleeds are focal lesions that result from a deposit of hemosiderin that presumably leaks out from damaged small brain vessels [74]. The prevalence of cerebral microbleeds was estimated as 17.8% in persons aged 60–69 years, and 38.3% in people over 80 years [74]. The presence of numerous microbleeds correlates with worse cognitive performance [77]. Moreover, lobar microbleeds are independently associated with executive dysfunction in patients with stroke or transient ischemic attacks [77]. Executive dysfunction is more prevalent in patients with cerebral microbleeds (38%) than other cognitive impairments [78].

A limitation of this study was to only include articles published in English. Despite this, very few manuscripts published in other languages were identified: in Portuguese [79] and in Norwegian [80]. Manuscripts reporting scales in languages other than English may have been published in journals not indexed in Medline, Pubmed and Google Scholar. This limits evaluation of validity and cross-cultural comparisons.

It is further noteworthy that literally all studies included in this review excluded patients with language impairments and those with severe physical disabilities. The findings on executive function performance summarized here are therefore not entirely translatable to the stroke population as a whole.

In a recent systematic review about cognitive rehabilitation after stroke, the authors discussed the importance of cognitive rehabilitation and the lack of evidence in this area [81]. Moreover, the review revealed that some of the cognitive domains (attention, spatial neglect and motor apraxia) can improve with rehabilitation, but this improvement is not long lasting. The authors also described the major limitations of evidence and the need for more investigations in this area with appropriate methodological standards.

## 5 Conclusion

Appropriate assessment of the patient with stroke is essential to provide better treatments. Awareness and quantification of impairments can enhance the ability to plan rehabilitation and optimize long-term care provision, in order to tailor treatments according to specific individual needs. This is particularly important for patients who might be able to return to work if their deficits in cognition are recognized. To attain this goal, adequate tools of evaluation, adapted to local cultural specificities, are required. There is a paucity of tools to reliably measure executive dysfunction after stroke, despite the fact that executive dysfunction is frequent. Specifically, there is a great need to develop appropriate tools for developing countries. In addition, limited information is available about the relation between executive dysfunction measured with valid scales and stroke lesions, white matter disease, microbleeds, as well as with other conditions that can be associated with stroke such as vascular cognitive impairment and depression. There are deep gaps about executive dysfunction in stroke, to be filled in research and in clinical practice.

## Funding

Annette Sterr, PhD is a Visitor Professor Neurostimulation Laboratory, Neurology Department at São Paulo University and received a “Science Without Boarders” scholarship (GRANT A 068/2013) from Conselho Nacional de Desenvolvimento Científico e Tecnológico (CNPq) and Coordenação de Aperfeiçoamento de Pessoal de Nivel Superior (CAPES). This investigation was supported by the Fogarty International Center of the National Institutes of Health under the NCD-LIFESPAN program (GRANT D71TW009132-01). Juliana Conti and Adriana Bastos Conforto received funding from the National Institutes of Neurological Disorders and Stroke (R01NS076348-01).

## Conflict of interest

The authors declare that there are no conflicts of interest.
